# Nano-LED induced chemical reactions for structuring processes

**DOI:** 10.1039/d0na00851f

**Published:** 2020-10-20

**Authors:** Martin Mikulics, Zdenĕk Sofer, Andreas Winden, Stefan Trellenkamp, Beate Förster, Joachim Mayer, Hilde Helen Hardtdegen

**Affiliations:** Ernst Ruska Zentrum (ER-C-2), Forschungszentrum Jülich GmbH D-52425 Jülich Germany; Jülich-Aachen Research Alliance, JARA, Fundamentals of Future Information Technology 52425 Jülich Germany; Department of Inorganic Chemistry, Institute of Chemical Technology Technická 5 Prague 6 Czech Republic zdenek.sofer@vscht.cz; Robert Bosch GmbH D-72760 Reutlingen Germany; Helmholtz Nanoelectronic Facility (HNF), Forschungszentrum Jülich GmbH D-52425 Jülich Germany; Ernst Ruska Zentrum (ER-C-1), Forschungszentrum Jülich GmbH D-52425 Jülich Germany

## Abstract

We present a structuring technique based on the initialization of chemical reactions by an array of nano-LEDs which is used in the near-field as well as in the far-field regime. In the near-field regime, we demonstrate first results with the nano-LED array for lithography using the photoresist DiazoNaphthoQuinone-(DNQ)-sulfonate for the fabrication of holes in the resist down to ∼75 nanometres in diameter. In contrast, the nano-LEDs can also be employed in the far-field regime to expose thin films of the monomer bisphenol A-glycidyl methacrylate (Bis-GMA) and to initialize polymerization locally. Photosensitive films were patterned and spherical cone-shaped three dimensional objects with diameters ranging from ∼480 nm up to 20 micrometres were obtained. The modification in the material as a result of the photochemical reaction induced *i.e.* by polymerization was confirmed by Raman spectroscopy. This structuring maskless technique has the potential to induce substantial changes in photosensitive molecules and to produce the desired structures from the tens of microns down to the nanometre scale.

## Abstract 2

We present a structuring technique based on the initialization of chemical reactions by an array of nano-LEDs which is used in the near-field as well as in the far-field regime.

## Graphical abstract

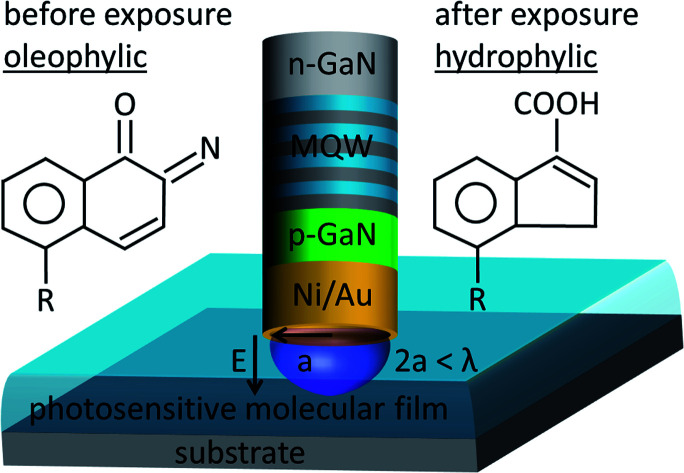

## Introduction

Rapid progress in the synthesis and fabrication of novel and nanometre scaled materials has led to a deeper insight into the fundamental physics and structure of matter. This development accelerates the information technological revolution in a way not seen before. The exploitation of these materials with their unique properties opens the way to a broad range of new devices resulting in applications and solutions in physics, chemistry, biology or medicine.^1–16^ However, before they can be fabricated industrially, structuring techniques have to be developed which allow the production of nanostructured materials reproducibly, in a short time and with a high yield. It is the precondition for successful future nanotechnology.

Conventionally, standard lithographic techniques^17–19^ are used for micro-structuring, which are now approaching their theoretical limits. Next improvements may only be reached by employing highly sophisticated optical techniques,^20,21^ special masks and short-wavelength excitation sources.^22,23^ In addition, other patterning techniques^24–29^ such as nanoimprint lithography are limited by material properties and time consuming steps. Although all presently used patterning techniques have been improved during the previous decades, the processing speed and efficiency desired still lag behind. Therefore there is a strong need to develop a suitably fast, flexible and efficient patterning and nanofabrication method.

Recently, we proposed an alternative lithography concept based on single photon sources and reported its realization as a proof of principle.^30,31^ Here in this work we present an approach based on so-called nano-LED Assisted Lithography (LEDALIT) which could significantly help increase and simplify the mass production of nanostructured objects and devices and help decrease production costs for a broad range of lithographic applications.

**Fig. 1:**
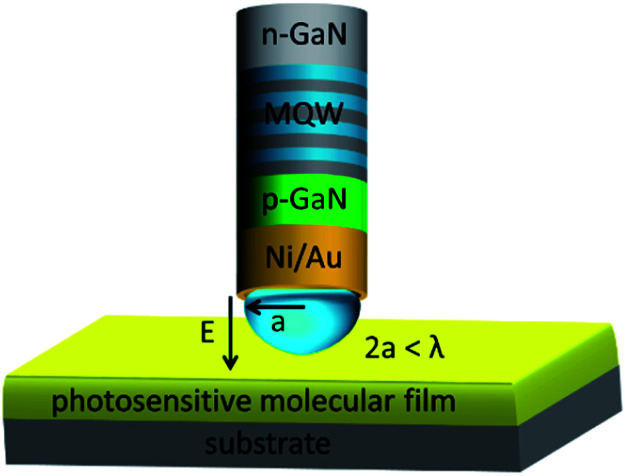
Principal schematics: nano-LED (n-GaN/MQW/p-GaN) element in the near-field regime to a photosensitive film generating an evanescent field. The fundamental goal of “near-field” LED assisted lithography (LEDALIT) is to create a sub-diffraction limited field distribution in a location favourable for a photochemical reaction.

**Fig. 2:**
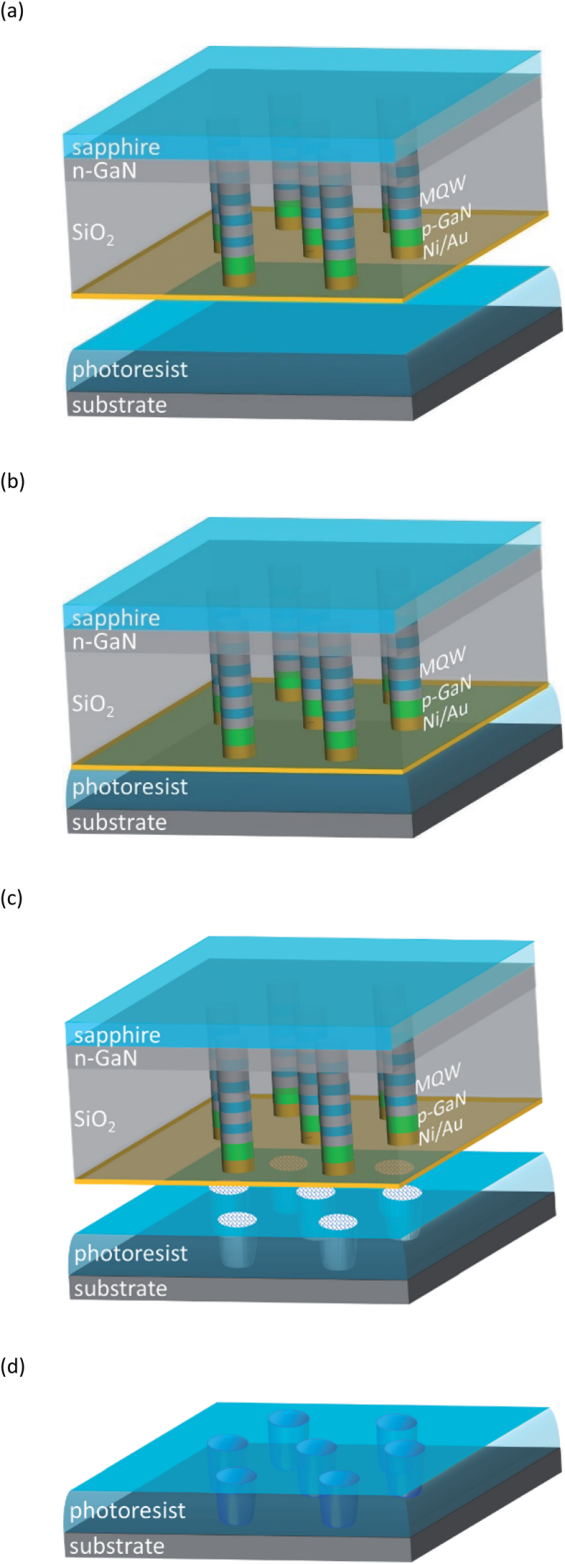
Schematics of the LEDALIT process: (a) individually addressable nano-LED elements are arranged in an array - in this case in a hexagonal pattern – on a chip; (b) nano-LEDs in the near-field regime to the photosensitive film producing locally an evanescent field and inducing photochemical reactions; (c) after exposure; (d) structured photoresist after development.

In the past, near-field fluorescence excitation and imaging have been demonstrated using single-photon point-like emitters^32–34^ such as nano-diamonds with nitrogen vacancies or quantum dot light emitting diodes (QDLEDs)^35^ integrated at the tip of a scanning probe. The technique takes advantage of the fact that objects considerably smaller than the diffraction limit are resolved in the near-field range.^36–38^ It has also been experimentally demonstrated, that chemical reactions can be induced in scanning near-field microscopy (SNOM).^39–42^ Our lithography approach based on nano-LED sources utilizes both these features – the near film distance and “few” photon emitters equipped with an aperture – to controllably induce photochemical reactions conventionally used for photoresists. The principal schematics is presented in Fig. 1. It shows a single nano-LED in the near-field range to the photo chemically sensitive film allowing the creation of a sub-diffraction limited field distribution in a location favourable for a photochemical reaction. The nano-LED sources are arranged in arrays and could be principally singularly addressable.^30^ The schematics of the lithography process is shown in Fig. 2. This concept allows for a very flexible process and renders mask patterning superfluous for structure definition.

Our approach, however, is not limited to nanometre scale systems and obtaining structures considerably smaller than the diffraction limit. It could be used also for the production of comparatively large, micrometre sized objects, if structuring were performed in the far-field regime. This feature of the technique is made possible by a precise control of nano-LED power and exposure time. If desired, a higher power and duration would allow an exposure of a large number of molecules, thus influencing the photoresist volume and thus enabling the realization of structures ranging from sub-micrometres up to several tens of micrometres using a single nano-LED element. In future, tunable arrays of singularly addressable nano-LED elements could have the potential to substantially increase the nano and micro device production efficiency and thus induce further progress in semiconductor industry.

## Experimental

### Materials and sample preparation

The deposition of the LED structure was carried out in a home-built metalorganic vapour phase epitaxy (MOVPE) reactor on *c*-plane sapphire substrates using the source compounds trimethylgallium, trimethylindium and ammonia at 200 hPa reactor pressure. At first, n-doped GaN was deposited in hydrogen ambient followed by a multiple quantum well structure (MQW) consisting of 2.5 nm In_0.1_Ga_0.9_N wells and 16 nm GaN barriers in nitrogen ambient. At last, 200 nm of p-doped GaN were deposited in hydrogen ambient. Well-established growth processes with respect to growth temperature were employed.^43–47^

**Fig. 3:**
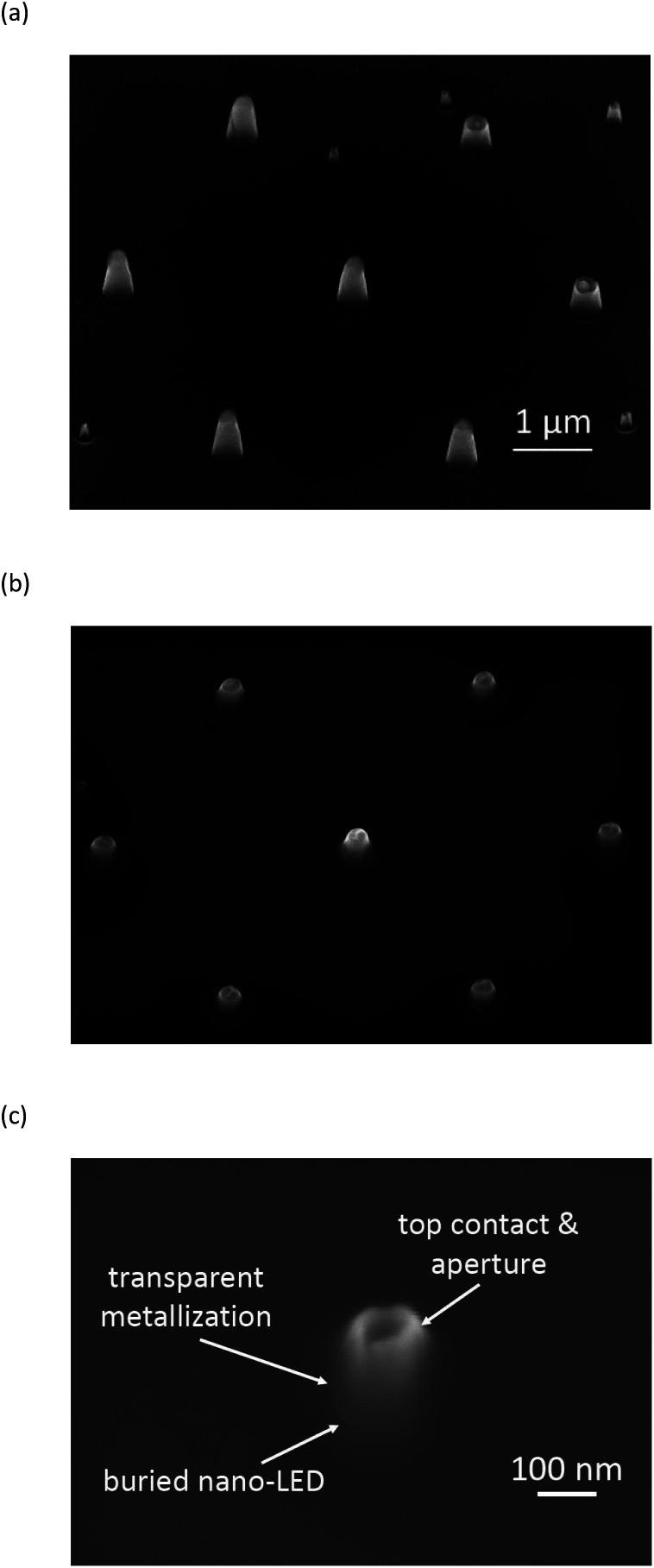
Nano-LEDs after Ar-IBE etching (a) and after covering them with a SiO_2_ layer (b), detail of buried nano-LED structure with an aperture (c). The nano-LEDs were positioned equidistantly to each other in hexagonal arrangement with a pitch of 3 μm.

After covering the LED layers with photoresist, holes were defined using electron-beam lithography and subsequent development. After the physical vapour deposition of Ni followed by a lift-off process, Ni caps were formed, which served as the mask for the subsequent Ar-ion beam etching process. Nano-LED structures were defined into arrays of 10 × 10 mm^2^. The nano-LEDs were positioned hexagonally with an individual spacing of 3 μm (see Fig. 3(a)). They exhibited a diameter of ∼100 nm. Further details have been reported elsewhere.^9,30^ All nanostructures were buried in a SiO_2_ layer (Fig. 3(b)) which served on the one hand as an isolation and on the other hand as a planarization layer for top contact processing. The detail of a buried nano-LED structure with an aperture is presented in Fig. 3(c).

The photoresist DiazoNaphthoQuinone-(DNQ)-sulfonate was chosen to produce the nanometre sized holes by exposing it to the nano-LEDs in the near-field regime. The exposure induces the reactions from the DNQ sulfonate to the respective carboxylic acid, which is soluble in a metal-free tetramethyl-ammonium hydroxide based developer.

For the production of micrometre sized structures, bisphenol A-glycidyl methacrylate (Bis-GMA) was spin-coated onto sapphire substrates. The nano-LEDs induced the chemical polymerization reaction.

### Characterization

The morphology of the group III nitride nanostructures and the structured photoresist was observed by conventional scanning electron microscopy (SEM, Zeiss LEO 1550, Gemini column). In contrast, the Bis-GMA monomer/polymer/sapphire substrate material system was investigated by environmental scanning electron microscopy (ESEM, Thermo Fisher Scientific, equipped with a field emission gun (FEG)). Due to the possibility of outgassing and to avoid any destruction that high vacuum may cause, the samples were characterized at a water vapour chamber pressure of 200 Pa and 20 kV acceleration voltage. In addition to top view images, cross-sectional optical microscope and SEM images were also taken of the nano-/microstructures induced by chemical reactions in the Bis-GMA to determine their shape. To this end, the samples were cooled down to liquid nitrogen temperature after the lithographical process and subsequently cut.

The electroluminescence of the nano-LEDs at room temperature was spectrally analyzed at 5 V bias voltage using a Renishaw spectrometer. A CCD camera was used to record the intensity.

The chemical reaction induced by the nano-LEDs in Bis-GMA was observed by performing Raman spectroscopy in backscattering geometry using a Renishaw inVia FSM-REFLEX confocal Raman spectrometer coupled with a 532 nm cw Nd:YAG laser. The spectra were recorded in the range from ∼1200 cm^−1^ to ∼2000 cm^−1^.

## Results and discussion

**Fig. 4:**
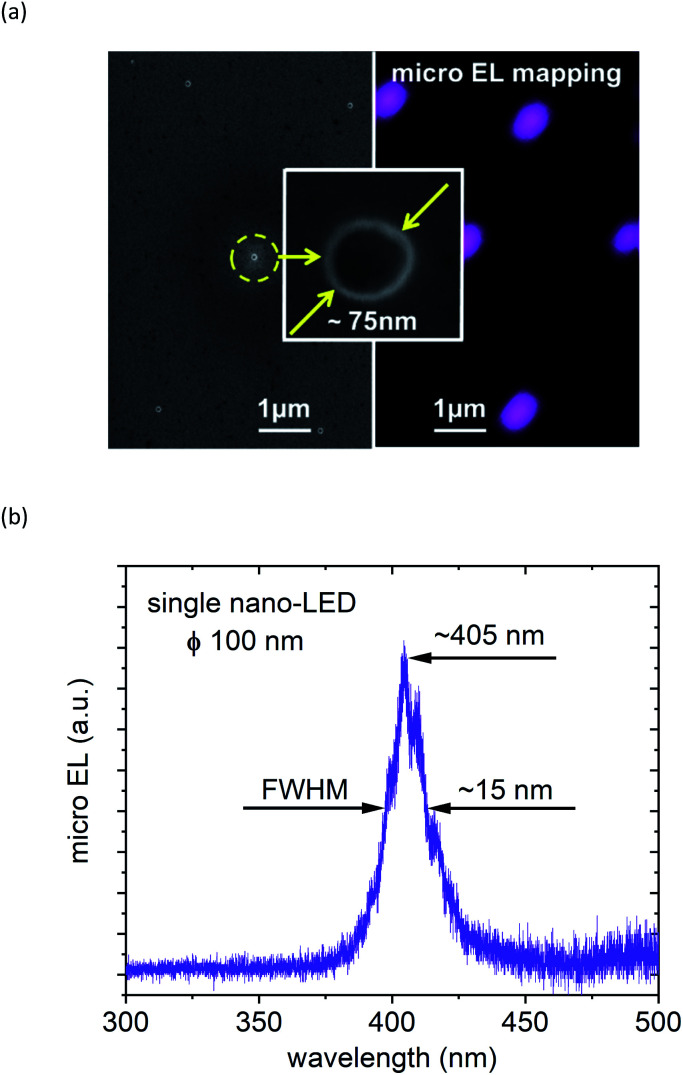
(a) Micro-EL mapping (right) and SEM images (left and centre) after nano-LED exposure in the near-field regime and the subsequent photoresist development process. The spacing between the “hole” nanostructures is 3 μm and the “hole” diameter is ∼70 ÷ 80 nm (inset) and (b) a representative electroluminescence spectrum from a single (100 nm in diameter) nano-LED structure. The nano-LED exhibits maximum emission at a central wavelength of ∼405 nm, which corresponds to a photon energy of ∼3.06 eV.

**Fig. 5:**
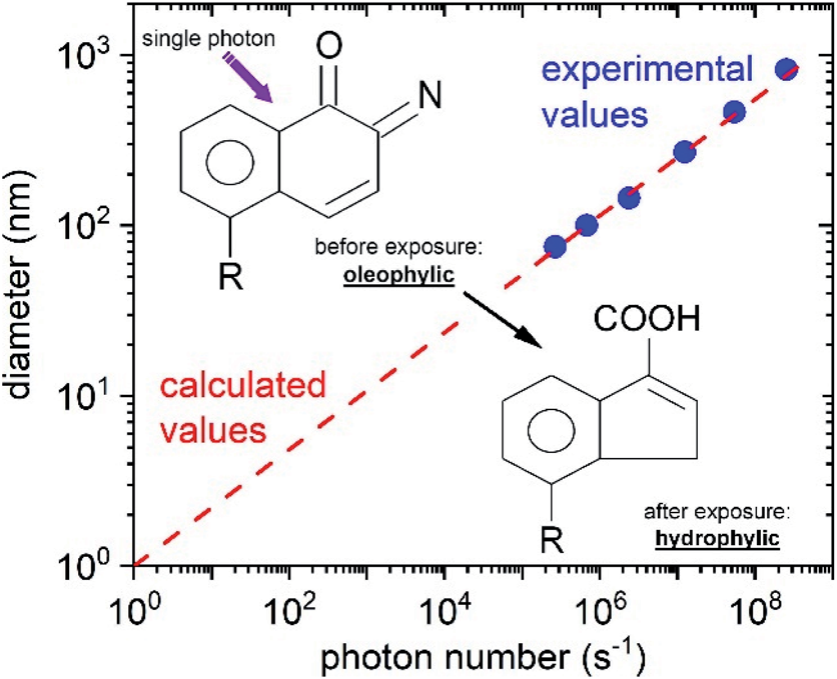
For the sake of comparison our experimental data (blue circles) obtained by the LEDALIT technique are compared with theoretical values^48^ (red dashed line) for a calculated “hole” diameter dependence as a function of photon number incident on the photoresist DNQ-sulfonate molecule. Inset: after the single photon interacts with the resist, a water-soluble carboxylic acid is formed.

The processed array of nano-LED structures was optically tested by micro-electroluminescence-mapping (see Fig. 4(a) right) at 5 V bias voltage and spectrally characterized. The luminescence intensity was recorded with a CCD camera. The electroluminescence intensity from all devices is in the same range, and is homogeneously distributed across the entire array, proving that we developed a highly reliable and reproducible technology for the production of nano-LEDs as the excitation source. The wavelength emitted from our single nano-LED structures is centred around 405 nm, which corresponds to a photon energy ∼3.06 eV (Fig. 4(b)) and is sufficient for the initialization of the photochemical reaction in the photoresist DiazoNaphthoQuinone(DNQ)-sulfonate.^49^ In the next step, the assembly of nano-LEDs was used as the source and the patterning mask simultaneously. After exposure and consecutive development in the tetramethylammonium hydroxide based developer, the attained structures (see Fig. 4(a) left and inset) in the photoresist were inspected by scanning electron microscopy (SEM). Dimensions down to ∼70 ÷ 80 nm were achieved using an exposure time of 3.2 s and development times varying between 12–15 s. A further decrease of the hole-diameter could be reached by reducing the applied nano-LED power and/or exposure time as has been theoretically predicted previously.^48^ This is well demonstrated in Fig. 5. We assume that the energy amount necessary for altering chemical bonds in our near-field process could be comparable to the process referred to as Single Photon Lithography (SPL).^30,31^ In SPL, theoretically in the ideal case, a single photon induces the reaction with just one photosensitive molecule, which is then the smallest possible structure size producible. Fig. 5 presents the calculated hole diameter dependence (red dashed line)^48^ as a function of photon number incident on the photoresist DNQ-sulfonate. In full analogy it can be expected, that the emission wavelength of the nano-LEDs will additionally affect the nano structure size and will be a variable, which can be fine-tuned to the respective structure dimensions desired.

**Fig. 6:**
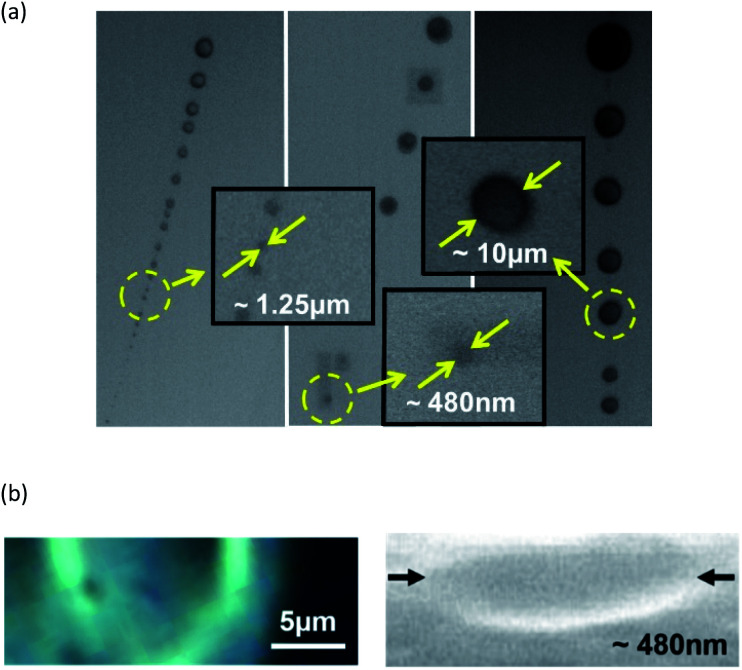
(a) ESEM images (plan view) of 3D structures patterned in bisphenol A-glycidyl methacrylate (Bis-GMA) after nano-LED (single device) exposure in the far-field regime. The 3D structure size depends on the exposure power and time applied by the nano-LED. Their diameters range from ∼480 nm up to close to 20 μm and (b) cross-sectional images by optical microscopy (left) and SEM (right) of two differently sized structures disclosing their spherical cone shape.

The main target of our technology was the next generation of sub-nanometre or molecular electronic applications primarily needed for highly secure communication systems and low energy consumption devices. However, the nano-LEDs can also be employed in the far-field exposure regime to induce photochemical reactions for the fabrication of larger scaled objects. To this end, thin films of bisphenol A-glycidyl methacrylate (Bis-GMA) – also known as Bowen monomer – were spin-coated onto sapphire substrates. In the next step the films were exposed to a single nano-LED emitting at ∼405 nm as is presented in Fig. 4(a). The photon energy of ∼3.06 eV (∼405 nm) is sufficient to initialize the polymerization process in Bis-GMA. The exposure leads to the patterning of the films and the structuring of spherical cone-shaped three dimensional objects with diameters ranging from ∼480 nm (using an exposure time set to 6.8 s) up to close to 20 μm as observable by environmental scanning electron microscopy (SEM, Fig. 6(a)) and by cross-sectional microscopy (Fig. 6(b)).

**Fig. 7:**
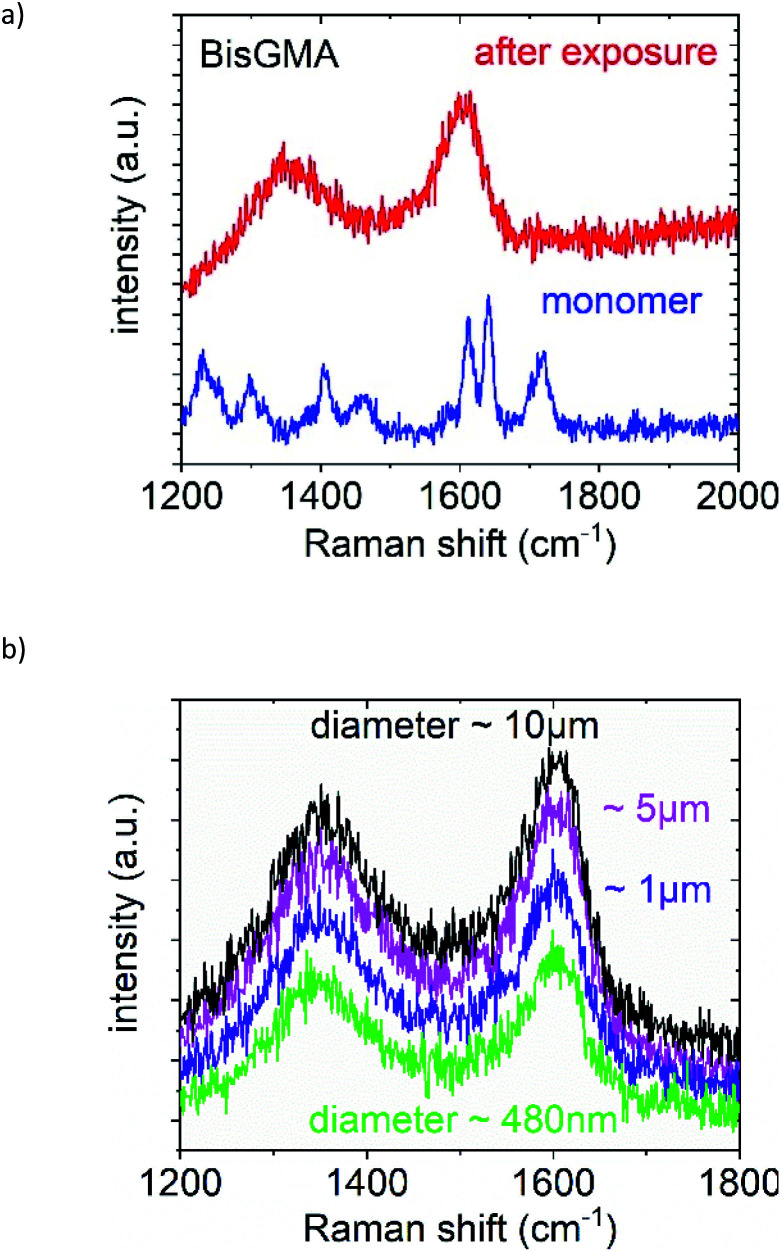
(a) Representative Raman spectra of Bis-GMA before (blue, monomer) and after (red, polymer) nano-LED exposure and (b) Raman spectra of Bis-GMA after the polymerization process initialized by a single nano-LED structure in the “far-field” regime; (b) spectra recorded for different cone shaped three dimensional structures with a diameter from ∼10 μm down to ∼480 nm (b).

The patterned sample was studied by Raman spectroscopy. The monomer Bis-GMA belongs to the family of the aromatic methacrylates exhibiting a well-characterized polymerization reaction the progress of which can be followed by Raman spectroscopy. Specifically the decrease in the intensity of the methacrylic C=O and the C=C stretching modes at ∼1716 cm^−1^ and ∼1638 cm^−1^, respectively, signalize the progress of the reaction. In contrast, the signature assigned to the aromatic C=C stretching mode stays constant during the polymerization reaction since the benzyl ring does not participate in the conversion from monomer to polymer.^50–54^ Two representative spectra of the Bis-GMA film are shown in Fig. 7(a) in the non-exposed (blue, monomer) and the nano-LED exposed area (red trace, corresponding to dark areas in the SEM micrograph). The Raman spectrum of the non-exposed area exhibits all signatures of the monomer molecule. The Raman spectrum of the exposed area *i.e.* of a patterned object is dominated by 2 broad peaks centred around 1350 cm^−1^ and ∼1600 cm^−1^. Here the C=O and the C=C stretching modes are no longer visible. This indicates that the nano-LED has induced a polymerization reaction in Bis-GMA resulting in the structuring of the film and the production of spherical cone-shaped objects.

Furthermore, Raman spectra were recorded of spherical cone-shaped three dimensional structures with different diameters created in the Bis-GMA layer. They are presented in Fig. 7(b). It is evident that all structures with a diameter from about 10 μm down to ∼480 nm exhibit an almost identical characteristic Raman signature. The Raman spectra for each nano-/microstructure diameter are obtained reproducibly with respect to their intensities within a tolerance better than ±2%. Hence, it is possible to identify also structures with dimensions below 500 nm in diameter, as well as their position with micro Raman spectroscopy. This is not always unambiguously possible by SEM inspection due to the difficulties related to contrast/resolution (see detail in Fig. 6(a)). They can be the result of beam-related contamination of the surfaces of the Bis-GMA monomer/polymer/sapphire substrate material system.

## Conclusions

In our work we demonstrated the potential of nano-LEDs to induce photochemical reactions for structuring processes. On the one hand they were applied in an array for lithography in the near-field regime using a conventional photo-resist. By controlling the exposure time, hole nanostructures with different dimensions down to ∼70 ÷ 80 nm were achieved controllably. Since the nano-LED arrays can consist of singularly addressable/driven elements in the future, structures and patterns can be designed with the desired size and geometry in a next step. On the other hand, a nano-LED was applied in the far-field regime to induce locally a polymerization reaction in a bisphenol A-glycidyl methacrylate film producing spherical cone-shaped objects in the film. These objects observed by SEM could be correlated by Raman spectroscopy to a new material most probably a polymer. It develops by the chemical reaction induced by the nano-LEDs in the Bis-GMA. By controlling the exposure power and time, objects were fabricated with dimensions between ∼480 nm up to several tens of micrometres. Structuring techniques based on nano-LEDs can simplify nanostructure production and increase flexibility greatly. By driving the LED elements accordingly, the pattern can be modified instantaneously without the dependence on classical masks. We also envision new prospects in the field of 2D and 3D printing allowing for the creation of many tens of micrometre and sub-micrometre 3D elements in micro-machining and mechatronics, microbiology, in the production of security labels and markers, as well as in molecular chemistry.

## Conflicts of interest

There are no conflicts to declare.

## Supplementary Material
